# Identification of piRNAs and piRNA clusters in the testes of the Mongolian horse

**DOI:** 10.1038/s41598-019-41475-9

**Published:** 2019-03-22

**Authors:** Bei Li, Xiaolong He, Yiping Zhao, Dongyi Bai, Gerelchimeg Bou, Xinzhuang Zhang, Shaofeng Su, Leng Dao, Rui Liu, Yuejiao Wang, Dugarjaviin Manglai

**Affiliations:** 1 College of animal science, Inner Mongolia Key Laboratory of Equine Genetics, Breeding and Reproduction, Scientific Observing and Experimental Station of Equine Genetics, Breeding and Reproduction, Ministry of Agriculture and Rural Affairs, Equine Research Center, Inner Mongolia Agricultural University, Hohhot, 010018 China; 2 Inner Mongolia Academy of Agricultural and Animal Husbandry Sciences, Hohhot, 010031 P. R. China

## Abstract

P-element induced wimpy testis-interacting RNAs (piRNAs) are essential for testicular development and spermatogenesis in mammals. Comparative analyses of the molecular mechanisms of spermatogenesis among different organisms are therefore dependent on accurate characterizations of piRNAs. At present, little is known of piRNAs in non-model organisms. Here, we characterize piRNAs in the Mongolian horse, a hardy breed that reproduces under extreme circumstances. A thorough understanding of spermatogenesis and reproduction in this breed may provide insights for the improvement of fecundity and reproductive success in other breeds. We identified 4,936,717 piRNAs and 7,890 piRNA clusters across both testicular developmental stages. Of these, 2,236,377 putative piRNAs were expressed in the mature samples only, and 2,391,271 putative piRNAs were expressed in the immature samples only. Approximately 3,016 piRNA clusters were upregulated in the mature testes as compared to the immature testes, and 4,874 piRNA clusters were downregulated. Functional and pathway analyses indicated that the candidate generating genes of the predicted piRNAs were likely involved in testicular development and spermatogenesis. Our results thus provide information about differential expression patterns in genes associated with testicular development and spermatogenesis in a non-model animal.

## Introduction

P-element induced wimpy testis (PIWI) proteins are a subfamily of Argonaute/PIWI proteins that are mainly expressed in the nuclei and cytoplasms of animal germ cells^1–4^. PIWI-interacting RNAs (piRNAs) are 26–32-nt PIWI-binding small noncoding RNAs that exhibit significant strand bias^5^. piRNA sequences are generated by repetitive sequences in the genome, which are distributed in clusters called piRNA clusters^6^. The distribution of piRNA clusters on different chromosomes is not uniform and is not proportional to the length of the chromosome^6^.

Cloning of miRNAs in mouse testis identified 381(~27 nt) putative piRNAs, while only 40 putative miRNAs(~22 nt) were identified in mouse oocytes, suggesting that piRNAs may have a specific role in the germlines of male mammals^7,8^. Two populations of piRNAs are expressed during the development and differentiation of mouse spermatogenic cells: one population of “classic” piRNAs that silences retrotransposons, and one population of “pachytene” piRNAs, generated from nontransposon intergenic regions primarily located in the pachytene spermatocytes^9^. The functions of this second population of piRNAs remain unknown. Pachytene piRNA characterization is therefore crucial for investigations of PIWI protein activity during mammalian spermatogenesis. In mice, pachytene piRNAs are involved in the elimination of large amounts of mRNA from the elongating spermatids^10^. In the germ cells of male mice, pre-pachytene piRNAs interact with the PIWI proteins MIWI2 and MILI1^11–14^. These proteins, in combination with transposons, retrotransposons, and other mobile genetic elements, ensure the normal development and differentiation of spermatogenic cells by inhibiting the activity of transposable elements at the epigenetic and post-transcriptional levels^15^. Deletion of either MIWI2 or MILI1 from the germ cells of fetal mice resulted in a significantly lower level of retrotransposon de novo DNA methylation compared to wildtype mice, indicating that the PIWI/piRNA pathway contributes to the horizontal transfer of silent transposable elements^16^.

Studies of piRNAs in horses and other non-model organisms are limited, thus hindering our understanding of the gene expression profiles and molecular mechanisms relating to deformation and maturation during spermatogenesis in such species. We selected the Mongolian horse for piRNA characterization, as this breed is particularly ancient, possibly expressing a phenotype ancestral to other Chinese, Japanese, and even Northern European horse breeds^17–19^. In addition, this breed has high endurance and is unusually hardy compared to other horses. Mongolian horses are capable of thriving in a harsh, cold, arid climate with poor grazing opportunities; horses reproduce in extreme conditions with little shelter and little provender^18^. A thorough understanding of spermatogenesis and reproduction in this breed may provide insights for the improvement of fecundity and reproductive success in other breeds. In particular, knowledge of how piRNA affect spermatogenesis in the Mongolian horse may provide a framework against which to compare other, less hardy horse breeds. Therefore, in this study we aimed to characterize the piRNAs from the testes of the Mongolian horse.

## Materials and Methods

### Sample collection

All experiments involving animals complied with the Animal Care Guidelines set out in the Declaration of the Institutional Animal Ethics Committee of the Inner Mongolia Agricultural University, Hohhot, Inner Mongolia, China, and were authorized by that committee. All possible care was taken to minimize animal suffering.

We received permission from the owner to geld six healthy male Mongolian horses in Xilingol League, Inner Mongolia, China. The ages of the horses were determined based on a physical examination of their teeth, and on information from the owner. Three colts (samples BS1-3) were between 11 and 13 months old, and three were between three and four years old (samples AS1-3). We surgically collected the testes of all six horses. Removed testes were stored in cryogenic vials with an RNA/DNA sample protector (Takara, Dalian, China). Small samples (~5 g) from the testes of each animal were immediately frozen in liquid nitrogen for quantitative real-time polymerase chain reaction (qPCR) analysis.

Anecdotal evidence suggests that male Mongolian horses are incapable of reproduction before 18 months^20,21^, and domesticated Mongolian horses are typical bred starting at age three (indicating sexual maturity)^20,21^. However, to confirm the sexual maturity of the colts from which the testes were taken, all testes were examined histologically.

### Construction of small RNA libraries and sequencing

We isolated total RNA from 100 mg of testicular tissue from each animal using TRIzol reagents (Invitrogen, Carlsbad, CA, USA). We tested each sample of total RNA for degradation and contamination with 1% agarose gels. We measured the purity and concentration of the total RNA samples with a NanoPhotometer spectrophotometer (Implen, Munich, Germany) and a Qubit RNA assay kit in a Qubit 2.0 Fluorometer (Life Technologies, Carlsbad, CA, USA), respectively. We measured the integrity of each total RNA sample with an Agilent RNA Nano 6000 assay kit on an Agilent Bioanalyzer 2100 system (Agilent Technologies, Palo Alto, CA, USA). We rejected any total RNA samples with a ratio of optical density (OD) at 260 nm to OD at 280 nm (OD260/280) < 1.7; a ratio of OD at 260 nm to OD at 230 (OD260/230) > 2.0; concentration < 300 ng/μL; or integrity < 7.

We used 3 μg total RNA from each sample as a template for library preparation with the NEBNext Multiplex Small RNA Library Prep Set for Illumina (NEB, Ipswich, MA, USA), following the manufacturer’s instructions. We built six libraries of small RNAs, one per sample. We used index codes to link each sequence to one of the six samples. In brief, the NEB 3′ SR adaptor was ligated to the 3′ ends of all miRNAs, siRNAs, and piRNAs. Following this ligation reaction, the SR RT primer was hybridized to an overabundance of 3′ SR adaptors (ensuring that some 3′ SR adaptors remained unligated), converting any remaining single-stranded DNA adaptors into double-stranded DNA molecules, and avoiding the formation of adaptor dimers. The 5′ SR adaptors were then ligated to the 5′ ends of the miRNAs, siRNAs, and piRNAs; as double-stranded DNA is not a substrate of T4 RNA Ligase 1, these were not ligated. We used RNase H- reverse transcriptase (NEB, Ipswich, MA, USA) to synthesize first-strand cDNA, and performed PCR using LongAmp Taq 2X Master Mix, SR Illumina primers, and index (X) primers. The PCR volume contained 2 μL of 10 µM SR Illumina primer, 2 μL of 10 µM index (X) primer, 2 μL of template DNA, and 25 μL of LongAmp Taq 2X Master Mix, made up to 50 μL with nuclease-free water. The cycling program was as follows: initial denaturation at 94 °C for 30 seconds; 30 cycles of 94 °C for 10 seconds, 50 °C for 30 seconds, and 65 °C for 50 seconds; and a final extension at 65 °C for 10 minutes. PCR products were separated on 8% polyacrylamide gels (100 V for 80 min). DNA fragments between 140 bp and 160 bp (the lengths of the small noncoding RNAs plus the 3′ and 5′ adaptors) were retrieved and dissolved in 8 μL elution buffer. cDNA library quality was evaluated using an Agilent Bioanalyzer 2100 System with High Sensitivity DNA Chips (Agilent Technologies, Palo Alto, CA, USA). The library preparations were sequenced on an Illumina Hiseq2500 platform, and 50 bp single-end reads were generated.

### Annotation of small RNAs

To produce clean sequence reads, we removed any reads containing polyA/T/G/C sections; any reads with 5′ adaptor contamination; any reads without 3′ adaptors; any reads without insert tags; and any reads of inferior quality (the quality values Q < 20 of the base number accounts for more than 30% of the total read reads.) from raw fastq. We confirmed that each small RNA mapped to a single annotation by performing annotations in a particular order, and removing small RNAs once they had been mapped. The order in which we performed annotations was known miRNAs, rRNAs, tRNAs, snRNAs, snoRNAs, repeats, genes, and novel miRNAs (Supplementary Table 1). The piRNA and piRNA cluster were analyzed based on data “repeat” and “other”.

### Identification of piRNAs and piRNA-generating genes

Determination of the best method for piRNAs in non-model organisms, such as the horse, is a difficult and unsolved problem. Reference piRNA sequences are available for only six model species (human, mouse, rat, *Drosophila*, zebrafish, and duckbill platypus; piRNABank; http://pirnabank.ibab.ac.in)^22^. Here, we used k-mer methods to identify piRNA sequences^23^. After aligning the repetitive sequences of identifying all piRNAs with the reference sequence, and then aligning the piRNA sequence without aligning the above repetitive sequence with the gene sequence of the reference genome, the piRNA-generating gene was obtained from the sam file based on alignment. The alignment software and parameters were bowtie (−v0–k1)^24^.

We analyzed the Gene Ontology (GO) of the all piRNA-generating genes using a GOseq-based Wallenius non-central hyper-geometric distribution^25^, which takes into account gene length bias. We used KOBAS^26^ to identify Kyoto Encyclopedia of Genes and Genomes (KEGG) pathways that were significantly enriched in the candidate piRNA generating genes.

### Identification and functional annotation of piRNA clusters

The bam alignment results of identifying all piRNAs on the reference genome sequence were obtained based on bowtie-v 0-k 1 alignment, and the coverage of piRNA on the reference sequence was obtained by using samtools depth. Only the coverage >= 2 piRNA was retained for subsequent analysis. The minimum length of piRNA cluster length is 200, and the threshold distance of interval length is 10000. We then determined the lengths of all piRNAs clusters as described above^5,15^. We then extended the range of each piRNA cluster to consider the sequences 2000 bp upstream and downstream using Perl script^5^. In this way, we detected neighboring gene piRNA clusters.

We measured the differential expression of each piRNA cluster between the mature and immature testes with DESeq^27,28^ in R v1.18.0^29^, setting padj < 0.05 as cutoffs. DESeq uses a model based on negative binomial distributions to identify differential piRNA cluster expression based on digital piRNA cluster expression data. Three biological replicates were performed for each sample.

### Validation of DESeq results

We validated the differential expression of eight randomly-selected piRNAs (uniq_1215060, uniq_4231549, uniq_1214880, uniq_1217459, uniq_7619, uniq_1214806, uniq_1447220, and uniq_1214700) with qPCR. To do this, we first extracted total miRNA from each of the six samples of previously frozen testicular tissue with a miRNeasy mini kit (Qiagen, Dusseldorf, Germany), following the manufacturer’s instructions. We resuspended the total miRNA in nuclease-free water and measured miRNA concentration with a NanoDrop 2000 (Thermo Fisher Scientific, Waltham, MA, USA). We used ~0.5 μg total miRNA as a template for the synthesis of first-strand cDNA with a miScript II RT Kit (Qiagen, Dusseldorf, Germany), following the manufacturer’s instructions. We diluted the cDNA to 0.1 μg/μL using RNAase-free water, following the instructions in the miRNeasy mini kit (Qiagen, Dusseldorf, Germany). Using the diluted cDNA as a template, we measured the expression of the eight piRNAs on an MX3000P Real-Time PCR System (Agilent Technologies, Palo Alto, CA, USA) with a miScript SYBR Green PCR Kit (Qiagen, Dusseldorf, Germany), following the manufacturer’s instructions. We used U6 as an internal reference gene to control for differences among samples. Although it has been suggested that U6 is not a suitable endogenous control^30^, our preliminary results suggested that U6 was reliable, as the cycle threshold values were uniform across samples, with a single smooth peak. We determined relative piRNA expression with the 2^−ΔΔCt^ method^31^. We measured significant differences in relative piRNA expression between the mature and immature horses using a one-way ANOVA in SAS v9.0 (SAS Institute Inc., Cary, NC, USA). We considered P < 0.05 statistically significant.

### Ethics approval and consent to participate

All procedures involving animals were approved and authorized by the Inner Mongolia Agricultural University. All experiments and methods were carried out according to guidelines and regulations of Inner Mongolia Agricultural University.

## Results

### Confirmation of sexual maturity and immaturity of testes

In the testis from colts BSM1-3, the seminiferous tubules had only single layers of germ cells, and were separated by interstitial cells (Fig. S1). Most of these cells were undifferentiated spermatogonia, although some spermatocytes were observed. No mature sperm were observed. Interstitial cells were present between seminiferous tubules. We thus concluded that horses BSM1-3 were sexually immature.

In the testis from colts ASM1-3, numerous germ cells in multiple layers were observed in the seminiferous tubule lumens. Mature sperm, spermatogonia, and spermatocytes were clearly visible (Fig. S1). We thus concluded that horses ASM1-3 were sexually mature.

### Putative piRNAs and candidate piRNA-generating genes

**Figure 1:**
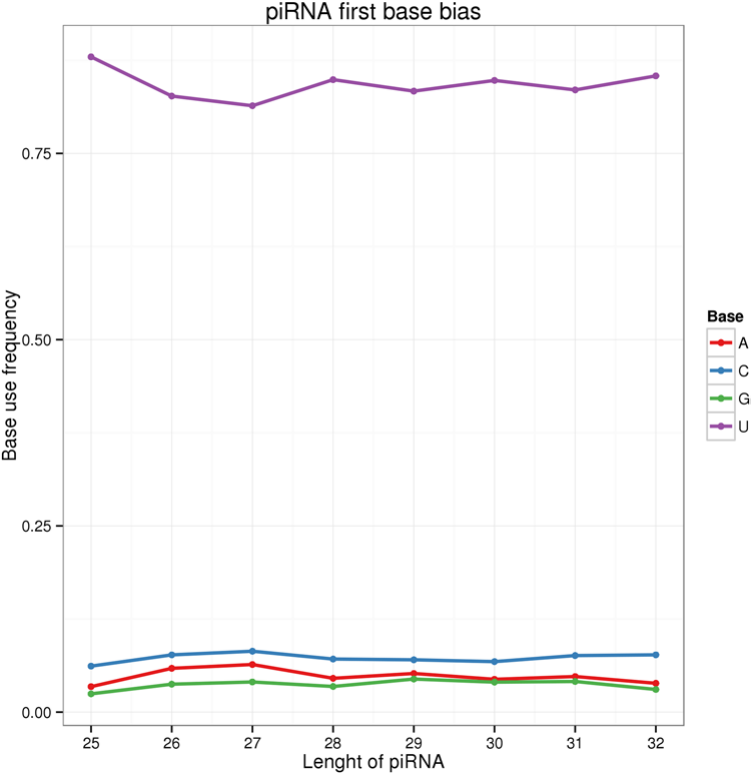
First base preference of piRNAs of different lengths.

We generated 843,017–1,287,314 raw reads for each of the six testicular samples (BS1: 843,017; BS2: 1,287,314; BS3: 941,342; AS1: 1,141,460; AS2: 1,126,573; and AS3: 1,141,984). We identified 4,936,717 unique putative piRNAs across all six libraries (Supplementary Table 2). Of these, 2,236,377 putative piRNAs were only expressed in the mature samples, and 2,391,271 putative piRNAs were only expressed in the immature samples. The putative piRNAs were 26–32 nt long. We observed a strong preference for uridine (U) at the 5′ end, and for adenine (A) at the 10th position (Fig. 1).

We predicted 30,639 generating genes for our 4,936,717 putative piRNAs (Supplementary Table 3). After eliminating repeated genes, unknown genes, and non-coding genes, 6921 known protein-coding genes remained. We classified the protein-coding genes related to spermatogenesis by the four types of proteins encoded: zinc finger proteins (targeted by piRNAs including uniq_2329918, uniq_2329932, and uniq_2329970), microtubule-associated proteins (targeted by piRNAs including uniq_2330366, uniq_2330761, and uniq_2337467), spermatogenesis-associated proteins (targeted by piRNAs including uniq_2338707, uniq_2339388, and uniq_2340251), and sperm-associated antigens (targeted by piRNAs including uniq_2343853, uniq_2343741, and uniq_2347690). We considered the genes encoding sperm-tail PG-rich repeat containing 2 (*STPG2*; generating genes by uniq_2341752) and meiosis 1-associated protein (*M1AP;* generated by uniq_2350120) spermatogenesis-related genes.

### Putative functions of identified piRNA clusters

**Figure 2:**
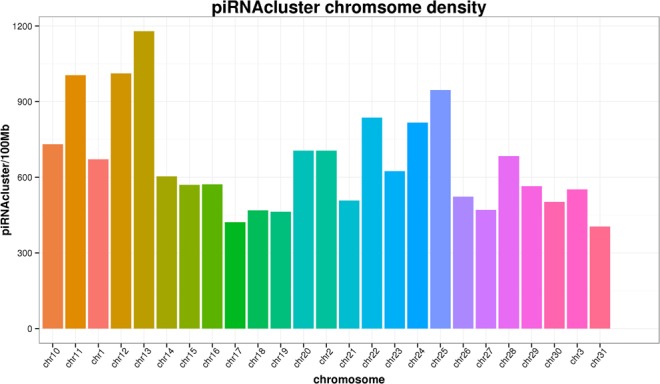
The distribution of piRNA clusters across chromosomes, showing that distributions were not uniform and were not proportional to the length of the chromosome.

We identified 7,890 piRNA clusters across all six libraries (Supplementary Table 4). Of these, 199 piRNA clusters were only expressed in the mature testes, and 1148 piRNA clusters were only expressed in the immature testes. The distribution of the piRNA clusters across chromosomes was non-uniform and was not proportional to the length of each chromosome (Fig. 2 and Supplementary Table 5).

**Figure 3:**
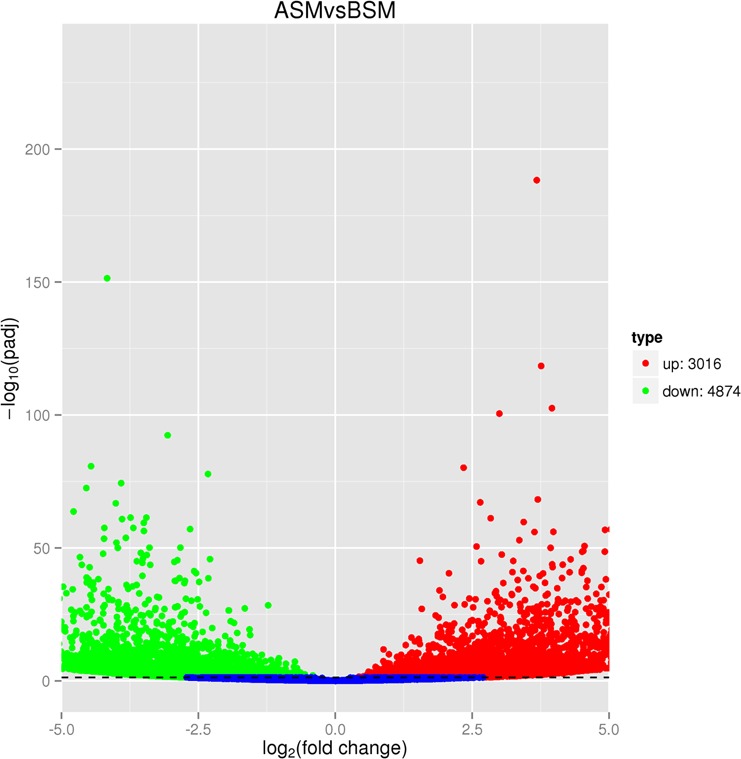
Significantly up- and downregulated piRNA clusters in mature testes as compared to immature testes.

Of the piRNA clusters shared by the two sets of samples, DESeq identified 3,016 that were upregulated in the mature testes as compared to the immature testes, and 4,874 that were downregulated in the mature testes as compared to the immature testes (Fig. 3). Several genes neighboring piRNAs clusters were significantly upregulated in the mature horses as compared to the immature horses, including spermatogenesis-associated 6 (*SPATA6*), meiotic double-stranded break formation protein 1 (*MEI1*), histone cluster 1, H2BA family (*HIST1H2BA*), testis-specific serine kinase 1B (*TSSK1B*), and centrosome- and basal body-related protein (*ALMS1)*. Our qPCR analysis validated the differential expression of the eight randomly-selected piRNAs in mature and immature horses (Supplementary Table 6), indicating that our DESeq analysis was reliable.

### Functional annotation of putative piRNA-generating genes

**Figure 4:**
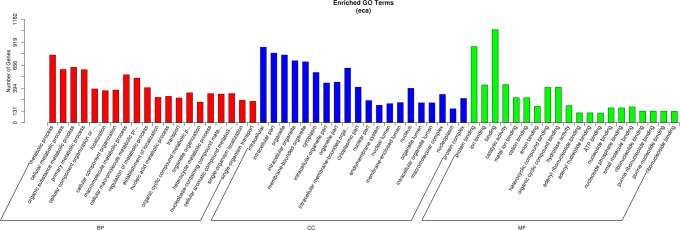
Gene ontology (GO) of the piRNA-generating genes. BP, biological process; CC, cellular component; MF, molecular function.

We identified 7,776 GO terms related to the all candidate piRNA-generating genes; 469 of these were significantly enriched (corrected P < 0.05; Fig. 4; Supplementary Table 7). We identified 273 significantly enriched GO terms related to biological processes, including metabolic processes (774 genes), cellular metabolic processes (608 genes), and organic substance metabolic processes (633 genes); 103 significantly enriched GO terms related to cellular components, including intracellular (863 genes), intracellular parts (796 genes), and organelles (774 genes); and 93 significantly enriched GO terms related to molecular function, including binding (1063 genes), protein binding (869 genes), and catalytic activity (435 genes). We identified 11 KEGG pathways significantly enriched in piRNA-generating genes (P < 0.05): focal adhesion, phosphatidylinositol signaling system, progesterone-mediated oocyte maturation, glutamatergic synapse, glycerolipid metabolism, dorsoventral axis formation, endocytosis, alanine, aspartate and glutamate metabolism, oocyte meiosis, adherens junction, and glycerophospholipid metabolism.

## Discussion

Consistent with previous results, the piRNAs that we identified in the Mongolian horse had a bias for U at the 5′ end, and in some cases the 10th base had a bias for A^32^; these bases are characteristic of miRNA sequences^32,33^. It is possible that these sequence features might be related to the ping-pong model of piRNA generation^33^. Piwi proteins are germline-specific argonaute proteins^33^ that play vital roles in piRNA biogenesis in *Drosophila* and Zebrafish^33–35^. Previous studies have identified five argonaute proteins in *Drosophila*: Ago1, Ago2, Ago3, Piwi, and Aubergine^36,37^. The piRNA sequences associated with Piwi and Aub are similar to the anti-sense strands of retrotransposons, while the piRNA sequences associated with Ago3 are similar to the sense strands^38^. Interestingly, piRNA sequences associated with Ago3 (sense strands) typically have an A at position 10, and piRNA sequences associated with Piwi and Aub (anti-sense strands) typically have a 5′ U^39^. Previous studies of cleavage activity in Piwi proteins suggest that this structure is typical of piRNAs^40^.

Our results indicated that the putative piRNAs we detected have similar features to previously described piRNAs, and are therefore likely to be true piRNAs. This suggested that primary piRNAs are tremendously heterogeneous group, characterized by a preference for a 5′ U, although the mechanistic explanation for this preference is unclear^39,40^. In PIWI proteins, the middle^39^ domain provides a structural basis for the enrichment of the 5′ U in miRNA sequences^39^. Indeed, MID domain structures in *Arabidopsis* argonaute proteins are enriched with small RNAs having distinct 5′ nucleotide biases for U, A, or cytosine (C)^39,40^

We identified 2,236,377 putative piRNAs only expressed in the mature samples, and 2,391,271 putative piRNAs only expressed in the immature samples. This suggested that piRNAs might play important role in spermatogenesis, especially at the early stages. We identified eight novel piRNAs with >500 reads in the immature testes, and eight novel piRNAs with >500 reads in the mature testes. Unfortunately, most of these novel piRNAs have not yet been annotated. This hampers our understanding of the function of these piRNAs in testicular development and spermatogenesis.

However, previous studies of piRNAs in humans indicate that piRNAs are likely to play a significant role in spermatogenesis^32,34^. For example, a previous study identified 20,121 piRNAs in normal human testis; of the piRNAs with >1000 reads, 12 mapped uniquely within testis developmental related gene 1 (TDRG1), while several others mapped in a sense orientation to an intron of cytochrome P450 family 19 subfamily A member 1 (CYP19A1)^41,42^. Both of these genes are associated with spermatogenesis: TDRG1 is a developmentally regulated testicular-specific gene, and CYP19A1 catalyzes androgens into estrogens^43^. Indeed, Flannigan^44^ identified significantly more piRNAs in the testicular tissues of normal men then in men with non-obstructive azoospermia (having Sertoli cells only), indicating that piRNAs are more abundant in sperm and spermatids, and thus probably play an important regulatory role in spermatogonia.

Here, although 4,936,717 putative piRNAs were screened, only 30,639 piRNA generating genes were predicted. Of predicted piRNA-generating genes, 6,969 were known protein coding genes. Two of these predicted piRNA-generating genes encode argonaute proteins: argonaute 1, RISC catalytic component (*AGO1*) and argonaute 3, RISC catalytic component (*AGO3*). Both *AGO1* and *AGO3* were significantly upregulated in the mature horse testes as compared to the immature horse testes, indicating that these piRNAs might be involved in spermatogenesis. Indeed, the AGO3 protein is required for RNA-mediated gene silencing (RNAi): it binds to short RNAs, such as miRNAs or siRNAs, to repress the translation of complementary mRNAs^45^. The transcriptional gene silencing (TGS) of promoter regions complementary to bound short antigene RNAs (agRNAs) also requires AGO3 AGO1 is a an important paralog of AGO3^45,46–48^. As typical argonaute proteins, AGO1 and AGO2 are also involved in miRNA-mediated gene management and siRNA-mediated mRNA degradation^45^. In contrast, AGO3, similar to the PIWI proteins and Aubergine, is associated with piRNAs, which primarily originate from transposon-rich clusters and play a pivotal role in transposon silencing^45^.

Two of the candidate generating genes encoded proteins in the PIWI clade: piwi-like RNA-mediated gene silencing 1 (*PIWIL1*), and piwi-like RNA-mediated gene silencing 2 (*PIWIL2*). Both *PIWIL1* and *PIWIL2* were upregulated in the mature testes as compared to the immature testes. *PIWIL1* is expressed in the testis, oocytes, and early embryos of cattle^49^, and PIWIL1 has been shown to inhibit the activity and movement of transposons during spermatogenesis by forming a complex with piRNAs^50^. *PIWIL1* is this critical for the maintenance of germline integrity^50^.

piRNAs may also be associated with other meiotic processes, such as the regulation of translation^51,52^ During piRNA biosynthesis, PIWIL2 plays a key role in the ping-pong amplification cycle, acting as a slicer-competent piRNA endoribonuclease that cleaves primary piRNAs; cleaved piRNAs are then loaded onto the slicer-incompetent PIWIL4^53^. *PIWIL2* is expressed in male as well as female germ cells^54^, indicating that this gene may function during oogenesis as well as spermatogenesis^15,49,52,55–58^.

We also identified several Tudor domain-containing genes (*TDRD*s) as candidate generating genes. Five *TDRD* genes (*TDRD1*, *TDRD5, TDRD9, TDRD12*, and *TDRD15*) were upregulated in the mature testes, as compared to the immature testes. During piRNA metabolism, TDRD proteins are associated with piRNA biogenesis^59^. In addition, TDRD proteins are critical for spermatogenesis because, like PIWIL1, they inhibit the activity and movement of transposons during spermatogenesis by forming piRNA and Piwi protein complexes, and thus maintain germline integrity^59^. The piRNA/PIWI multiprotein complexes are involved in secondary piRNA metabolic processes^60^, acting via the PIWI-EXD1-Tdrd12 (PET) complex during the PIWIL4 piRNA loading that is triggered by PIWIL2 slicing^58^.

Some piRNA-generating genes with spermatogenesis were detected, such as zinc finger protein genes, microtubule associated protein genes, spermatogenesis-associated protein gene, and sperm-associated antigens. These genes had a strong relationship with spermatogenesis^61–64^. Their specific functions should be determined in future research.

Therefore, all candidate piRNA-generating genes identified here were associated with the regulation of piRNA generation. Primary piRNA transcripts are generated from the transposon regulatory regions of heterochromatin^33^. These primary piRNA transcripts, associated with both Piwi proteins and Aub, are antisense and complement the transposon transcripts^33^. Piwi and Aub cleave the target transposon transcripts at 10–11 nt from the 5′ end of the antisense piRNA, generating Ago3-associated sense piRNAs^38^. Ago3 recognizes the piRNA cluster transcripts, and generates more Piwi/Aub-associated antisense strand piRNAs^38,65^. The regulation of piRNAs and their generating genes in the Mongolian horse is a target of our future research.

Of the 7,890 piRNA clusters we identified, 3,016 were upregulated in mature testes as compared to immature testes, and 4,874 were downregulated in mature testes as compared to immature testes. piRNA transcriptomes might be strongly adaptive because piRNA clusters incorporate exogenous DNA to provide the substrate for new antisense piRNAs^66^. In propagating transposons, piRNA promote adaptive immunity to selfish DNA^67^. However, mRNAs may be reverse transcribed and reintegrated into existing piRNA clusters, generating pseudogenes that encode piRNAs; these piRNAs may regulate genes^68^. Certain loci may also encode gene-regulating piRNAs on one strand, and genes encoding functional proteins on the other strand^69^.

We were able to annotate some of the genes neighboring the identified piRNA clusters. In particular, SPATA6 is required in late spermatogenesis for the formation of the link connecting the head and flagellum of the sperm^66,70^, while MEI1 is required for the formation of meiotic spindles in female germline cells, and may perform a similar function in males^71,72^.

The GO terms significantly enriched among the candidate piRNA-generating genes included several that are closely related to spermatogenesis: regulation of microtubule-based processes, cell cycle, cellular biosynthetic processes and RNA biosynthetic processes, cell development, sex differentiation, regulation of cell proliferation, positive regulation of mononuclear cell proliferation, and epigenetic regulation of cell growth.

Our functional assessments of the candidate generating genes of the piRNA clusters indicated that the piRNAs and piRNA clusters identified in the Mongolian horses were strongly associated with spermatogenesis. However, the details of these relationships warrant further study. Indeed, we are currently conducting functional verification studies of several piRNAs and their generating genes.

## Supplementary information

The title page and supplemental files legend

Dataset 1

Dataset 2

Dataset 3

Dataset 4

Dataset 5

Dataset 6

Dataset 7

## Data Availability

All raw and processed non-coding RNA profiles have been submitted to the NCBI as a GEO dataset (GEO: GSE100852).
