# Supplementary material for: Identification of piRNAs and piRNA clusters in the testes of the Mongolian horse
Source: Sci Rep. 2019 Mar 22;9:5022. doi: 10.1038/s41598-019-41475-9 (PMC6430771; doi:10.1038/s41598-019-41475-9)
Supplement: Supplementary file 1 — The title page and supplemental files legend [file 41598_2019_41475_MOESM1_ESM.pdf]

Identification of piRNAs and piRNA clusters in the testes of the Mongolian horse

Bei Li*, Xiaolong He[#], Yiping Zhao*, Dongyi Bai*, Gerelchimeg Bou*, Xinzhuang Zhang*, Shaofeng Su*, Leng Dao*, Rui Liu*, Yuejiao Wang*, Dugarjaviin Manglai*[*]

*College of animal science; Inner Mongolia Key Laboratory of Equine Genetics, Breeding and Reproduction; Scientific Observing and Experimental Station of Equine Genetics, Breeding and Reproduction, Ministry of Agriculture and Rural Affairs; Equine Research Center, Inner Mongolia Agricultural University, Hohhot 010018, China

#Inner Mongolia Academy of Agricultural and Animal Husbandry Sciences, Hohhot 010031, PR China

Abstract: P-element Induced WImpy testis-interacting RNAs (piRNAs) are essential for testicular development and spermatogenesis in mammals. Comparative analyses of the molecular mechanisms of spermatogenesis among different organisms are therefore dependent on accurate characterizations of piRNAs. At present, little is known of piRNAs in non-model organisms. Here, we characterize piRNAs in the Mongolian horse, a hardy breed that reproduces under extreme circumstances. A thorough understanding of spermatogenesis and reproduction in this breed may provide insights for the improvement of fecundity and reproductive success in other breeds. We

[*]Correspondence author: Dugarjaviin Manglai
Tel: +86 13848712222;
E-mail:dmanglai@163.com

identified 4,936,717 piRNAs and 7,890 piRNA clusters across both testicular developmental stages. Of these, 2,236,377 putative piRNAs were expressed in the mature samples only, and 2,391,271 putative piRNAs were expressed in the immature samples only. Approximately 3,016 piRNA clusters were upregulated in the mature testes as compared to the immature testes, and 4,874 piRNA clusters were downregulated. Functional and pathway analyses indicated that the candidate generating genes of the predicted piRNAs were likely involved in testicular development and spermatogenesis. Our results thus provide information about differential expression patterns in genes associated with testicular development and spermatogenesis in a non-model animal.

Keywords: Mongolian horse, piRNAs, piRNA cluster, testicular development, spermatogenesis

Supplemental Files Legends

FigS1. Representative images of the histological testicular sections from all three younger horses (11–13 months old; BSM; left panels) and all three older horses (3–4 years old; ASM; right panel). Cells in BSM testis were primarily undifferentiated spermatogonia, although some spermatocytes were observed. No mature sperm were observed. Interstitial cells existed between seminiferous tubules. In the ASM testes, numerous germ cells in multiple layers were observed in the lumens of the seminiferous tubules. Sperm, spermatogonia, and spermatocytes were clearly visible. Scale bars: 10 μm.

Table1. the number of kinds of sRNAs

Note:

Total: Number of sRNAs for comparison sequences in each sample

known_miRNA: The number and proportion of sRNAs were compared with known miRNAs in each sample

rRNA/tRNA/snRNA/snoRNA: The number and proportion of sRNA were compared with rRNA/tRNA/snRNA/snoRNA in each sample.

Repeat: The number and proportion of sRNA were compared to repeat in each sample.

NAT: The number and proportion of sRNA were compared with NAT gene in each sample.

novel_miRNA： The number and proportion of sRNA were compared with novel

miRNA in each sample.

TAS：The number and proportion of sRNA were compared with TAS gene in each sample.

exon:+/exon:-/intron:+/intron:-：The number and proportion of sRNA were compared with the exon/intron plus and minus strand.

other：The number and proportion of sRNA were compared to reference sequences, but were not compared in known miRNAs, ncRNA, repeat, NAT, novel miRNAs, TAS and gene exons and introns of each samples.

Table2. Expression of novel piRNAs.

Note: (1) piRNA ID. (2) piRNA reads across the six samples. (3) piRNA sequence.

Table3. The relationship between piRNAs and piRNA-generating genes.

Note: (1) piRNA ID.(2) Ensembl_Gene_ID: Ensembl ID of the piRNA-generating gene.(3) Associated Gene Name: piRNA-generating gene name.(4) Description: description of the piRNA-generating gene

Table4. Differential expression of piRNA clusters in mature and immature horse testes.

Note: (1) Cluster: piRNA cluster id. (2) ASM_readcount: corrected ASM read count. (3) BSM_readcount: corrected BSM read count. (4) Log2. fold_change: log2(ASM read count/BSM read count). (5) p-value. (6) q-value: corrected p-value.

Table5. The position of the piRNA clusters on the chromosome.

Note: (1) piRNA cluster ID. (2) Position: position of cluster on the chromosome

Table6. Differential expression of eight novel piRNAs, as quantified by qPCR.

Table7. The gene ontology (GO) of the piRNA-generating genes.

Note: (1) GO accession: GO accession number. (2) Description: GO gene function. (3) Term type: GO term type. (4) Significance of over-representation: statistical significance of the enrichment level. (5) Corrected p-value: corrected p-value.(6) DEG item: the number of piRNA-generating genes associated with the GO term (7) DEG list: The annotated number of piRNA generating genes associated with the GO term.(8) Bg item: All genes associated with this GO term. (9) Gene_names: the Ensembl IDs of the piRNA-generating genes associated with this GO term.

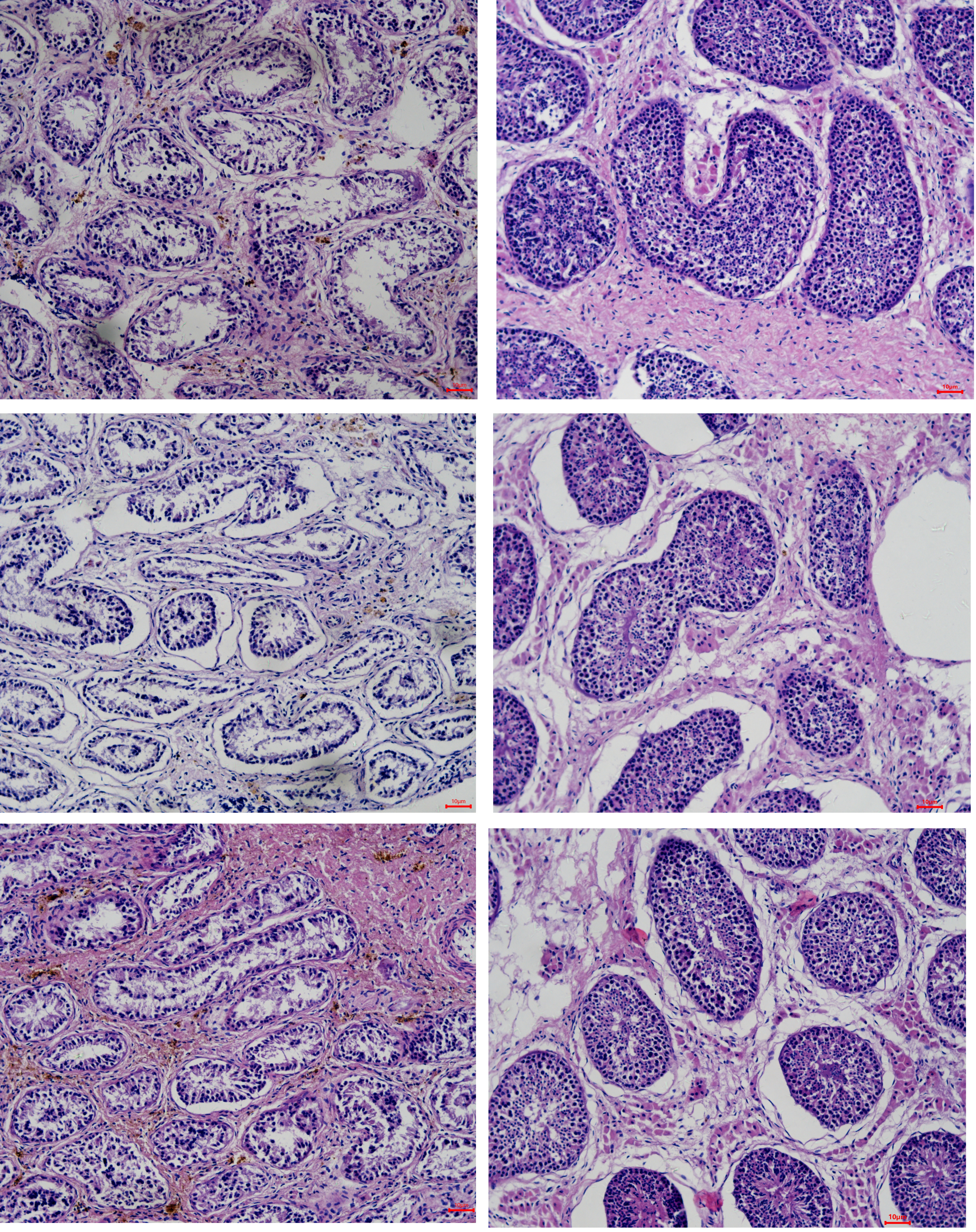

10μm
10μm
10μm
10μm
10μm
10μm
